# Simultaneous laparoscopic nephroureterectomy and robot-assisted anterior pelvic exenteration with intracorporeal ileal conduit urinary diversion: step-by-step video-illustrated technique

**DOI:** 10.1590/S1677-5538.IBJU.2020.1006

**Published:** 2021-01-20

**Authors:** Éder Silveira Brazão, Daniel Gomes Coser, Rafael Ribeiro Meduna, Walter Henriques da Costa, Stênio de Cássio Zequi

**Affiliations:** 1 AC Carmargo Cancer Center Departamento de Urologia São PauloSP Brasil Departamento de Urologia, AC Carmargo Cancer Center, São Paulo, SP, Brasil

## Abstract

**Introduction:**

One of the most remarkable characteristics of urothelial carcinomas is multifocality. However, occurrence of synchronous bladder cancer and upper urinary tract urothelial cancer (UTUC) is exceptional. Minimally invasive approach for these synchronous tumors was just occasionally reported (1-4). The aim of this video article is to describe step-by-step the technique for simultaneous laparoscopic nephroureterectomy and robot-assisted anterior pelvic exenteration with intracorporeal ileal conduit urinary diversion (ICUD). Patients and methods: A 66-year-old female presented with synchronous BCG refractory non-muscle invasive bladder cancer and a right-side UTUC. She was a former smoker and had previously been submitted to multiple transurethral resections of bladder tumor, BCG and right distal ureterectomy with ureteral reimplant. We performed a simultaneous laparoscopic right nephroureterectomy and robot-assisted anterior pelvic exenteration with totally intracorporeal ICUD. Combination of robot-assisted and pure laparoscopic approaches was proposed focusing on optimization of total operative time (TOT).

**Results:**

Surgery was uneventful. TOT was of 330 minutes. Operative time for nephroureterectomy, anterior pelvic exenteration and ICUD were 48, 135, 87 minutes, respectively. Estimated blood loss was 150mL. Postoperative course was unremarkable and patient was discharged after 7 days.

Histopathological evaluation showed a pT1 high grade urothelial carcinoma plus carcinoma in situ both in proximal right ureter and bladder, with negative margins. Twelve lymph nodes were excised, all of them negative.

**Conclusion:**

In our preliminary experience, totally minimally invasive simultaneous nephroureterectomy and cystectomy with intracorporeal ICUD is feasible. Pure laparoscopic approach to upper urinary tract may be a useful tactic to reduce total operative time.

**Available at:** http://www.intbrazjurol.com.br/video-section/20201006_Brazao_et_al

**Int Braz J Urol. 2021; 47 (Video #17): 1072-3**

## ABBREVIATIONS

ICUD: Ileal Conduit Urinary Diversion
UTUC: Upper urinary Tract Urothelial Carcinoma
TOT: Total Operative Time

## References

[1] 1. Berglund RK, Matin SF, Desai M, Kaouk J, Gill IS. Laparoscopic radical cystoprostatectomy with bilateral nephroureterectomy: initial report. BJU Int. 2006; 97:37-41. 10.1111/j.1464-410X.2005.05897.x 16336325

[2] 2. Barros R, Frota R, Stein RJ, Turna B, Gill IS, Desai MM. Simultaneous laparoscopic nephroureterectomy and cystectomy: a preliminary report. Int Braz J Urol. 2008; 34:413-21. 10.1590/s1677-55382008000400003 18778492

[3] 3. Ou YC, Yang CR, Yang CK, Cheng CL, Hemal AK. Simultaneous robot-assisted nephroureterectomy and cystectomy in patients with uremia and multifocal urothelial carcinoma. J Endourol. 2011; 25:979-84. 10.1089/end.2010.0602 21476927

[4] 4. Buse S, Hach CE, Alexandrov A, Mager R, Haferkamp A. Simultaneous en-bloc robot-assisted radical cystectomy and nephro-ureterectomy: technique description, outcomes, and literature summary. J Robot Surg. 2016; 10:315-322. 10.1007/s11701-016-0600-1 27153839

